# Association of CT-Based Delta Radiomics Biomarker With Progression-Free Survival in Patients With Colorectal Liver Metastases Undergo Chemotherapy

**DOI:** 10.3389/fonc.2022.843991

**Published:** 2022-05-27

**Authors:** Shuai Ye, Yu Han, XiMin Pan, KeXin Niu, YuTing Liao, XiaoChun Meng

**Affiliations:** ^1^The Sixth Affiliated Hospital, Sun Yat-sen University, Guangzhou, China; ^2^The Third Affiliated Hospital, Sun Yat-sen University, Guangzhou, China; ^3^GE Healthcare Pharmaceutical Diagnostics, Guangzhou, China

**Keywords:** radiomics, computed tomography, progression-free survival, colorectal liver metastases, chemotherapy

## Abstract

Predicting the prognosis of patients in advance is conducive to providing personalized treatment for patients. Our aim was to predict the therapeutic efficacy and progression free survival (PFS) of patients with liver metastasis of colorectal cancer according to the changes of computed tomography (CT) radiomics before and after chemotherapy.

**Methods:**

This retrospective study included 139 patients (397 lesions) with colorectal liver metastases who underwent neoadjuvant chemotherapy from April 2015 to April 2020. We divided the lesions into training cohort and testing cohort with a ratio of 7:3. Two - dimensional region of interest (ROI) was obtained by manually delineating the largest layers of each metastasis lesion. The expanded ROI (3 mm and 5 mm) were also included in the study to characterize microenvironment around tumor. For each of the ROI, 1,316 radiomics features were extracted from delineated plain scan, arterial, and venous phase CT images before and after neoadjuvant chemotherapy. Delta radiomics features were constructed by subtracting the radiomics features after treatment from the radiomics features before treatment. Univariate Cox regression and the Least Absolute Shrinkage and Selection Operator (LASSO) Cox regression were applied in the training cohort to select the valuable features. Based on clinical characteristics and radiomics features, 7 Cox proportional-hazards model were constructed to predict the PFS of patients. C-index value and Kaplan Meier (KM) analysis were used to evaluate the efficacy of predicting PFS of these models. Moreover, the prediction performance of one-year PFS was also evaluated by area under the curve (AUC).

**Results:**

Compared with the PreRad (Radiomics form pre-treatment CT images; C-index [95% confidence interval (CI)] in testing cohort: 0.614(0.552-0.675) and PostRad models (Radiomics form post-treatment CT images; 0.642(0.578-0.707), the delta model has better PFS prediction performance (Delta radiomics; 0.688(0.627-0.749). By incorporating clinical characteristics, CombDeltaRad obtains the best performance in both training cohort [C-index (95% CI): 0.802(0.772-0.832)] and the testing cohort (0.744(0.686-0.803). For 1-year PFS prediction, CombDeltaRad model obtained the best performance with AUC (95% CI) of 0.871(0.828-0.914) and 0.745 (0.651-0.838) in training cohort and testing cohort, respectively.

**Conclusion:**

CT radiomics features have the potential to predict PFS in patients with colorectal cancer and liver metastasis who undergo neoadjuvant chemotherapy. By combining pre-treatment radiomics features, post-treatment radiomics features, and clinical characteristics better prediction results can be achieved.

## Introduction

Globally, colorectal cancer ranks as the third most common type of cancer but ranks second in terms of mortality (1). Approximately 50% to 60% of patients diagnosed with colorectal cancer will develop colorectal metastases (2–4) and the liver is the most common location for of metastasis (5). Colorectal metastasis is usually metachronous (after local colorectal cancer treatment) (5). An estimated 20%–34% of patients with colorectal cancer present with synchronous liver metastases (6, 7). Synchronous colorectal liver metastases (CRLM) patients tend to have a poor prognosis with a reported 1-year survival less than 30% and a 5-year survival less than 5% if untreated. The 5-year survival rates for the selected group that can undergo curative surgical resection can be up to 60% (8). For patients with CRLM, complete surgical resection of all metastases is considered to be the only curative method (9, 10). However, 80%–90% of the patients with CRLM cannot receive curative surgical resection due to either the tumor being too large or medical conditions accompanying the disease (3, 6, 11–16). Patients with unresectable CRLM have indications for palliative systemic treatment and will undergo neoadjuvant chemotherapy (14, 17). Depending on the therapeutic effect of neoadjuvant chemotherapy, patients may receive surgical resection or local treatment (18). Predicting the prognosis of patients with neoadjuvant chemotherapy in advance will help doctors in making treatment decisions or adjustments.

Computerized tomography (CT) imaging plays a vital role in the diagnosis and efficacy prediction of CRLM. To date, the main content of imaging prediction includes the visual assessment of the lesion size and morphological changes in response to treatment. However, the information obtained from CT is limited because it mainly relies on visual assessment. Fortunately, recent studies have shown that texture analysis enhances the interpretation of CT images, which may reveal underlying tumor biology (19). It can directly extract biological data from radiographic images without invasive operations, thereby, saving cost, time, and avoiding most of all risk to the patient. Texture analysis of CT images involves a computational process, which can spatially quantify the voxel of CT images and effectively correlate the structural features of tumors with the voxel features of CT images (20). In patients with CRLM, texture analysis has been studied using CT data. To date, two main settings have been explored: one group of studies focused on the intralesional texture of the liver metastases itself, which was found to be significantly correlated with the response to chemotherapy, as well as with patient survival (21–24). Other studies focused not on the texture of the metastases but on that of the surrounding liver parenchyma and showed that diffuse parenchymal textural changes may hold promise as a prognostic marker to assess and even predict the occurrence of metastatic disease in the liver (23, 24). Although the texture of metastatic liver cancer and their surrounding parenchyma has been studied, to our knowledge, whether the differences in the focal image texture before and after treatment can predict the curative efficacy has not been reported. This will be an interesting study that will provide valuable insights into the relationship between novel imaging biomarkers and underlying tumor behavior.

The purpose of this study is to combine CT imaging and clinical characteristics to study the image texture changes of colorectal liver metastases before and after treatment, in the hope of helping to predict the efficacy of chemotherapy, and thus contributing to treatment decision-making.

## Methods

### Patients

The retrospective study included patients with liver metastases from colorectal cancer diagnosed at the Sixth Affiliated Hospital of Sun Yat-sen University from April 2015 to April 2020. The TNM Classification of Malignant Tumors (TNM) stages, pathological types and differentiation, chemotherapy regimen, immunohistochemistry, gene detection and laboratory results (alanine transaminase, aspartate transaminase, glutamine transaminase, alkaline phosphatase, total bilirubin, direct bilirubin, total bile acid, alpha fetoprotein, carcinoembryonic antigen) of the patients were reviewed. All patients met the following inclusion criteria: (a) pathologically confirmed colorectal adenocarcinoma, (b) first-time and untreated patients, (c) CT plain scan and enhanced examination were performed, and (d) reexamined within 3 months after chemotherapy. The exclusion criteria for this study were: (a) patients who had received neoadjuvant chemotherapy or radiotherapy before surgery and (b) first-time patients without liver metastases. A total of 139 patients with an average age of 57 ± 10 years were included.

### Treatments and Follow-Up

All patients underwent neoadjuvant chemotherapy and underwent imaging follow-up. The time of our study began with CT plain scan and contrast-enhanced CT examination at the first diagnosis. After chemotherapy, patients were followed up every 2-4 months until the progression of liver metastasis, other distant metastasis, the last follow-up date, or death occured. Progression free survival (PFS) was measured in months from the date of first diagnosis to the first date of local recurrence or progression, distant metastasis, last follow-up date, or death, whichever came first. Overall survival (OS) was measured in months from local recurrence or progression to the date of death or the last follow-up. The last follow-up date was February 5, 2021.

### CT Image Acquisitions

A Toshiba 640-slice CT scanner (Toshiba Medical Systems, Tokyo, Japan) was used to perform contrast-enhanced CT examinations at a tube voltage of 120 kV, automatic tube current modulation, 0.814 pitch, and 0.5 mm reconstruction section thickness. All patients received intravenous injection of a contrast agent (Iopromide, Bayer Healthcare, 370 mg / ml, 1.3-1.5ml / kg, and a injection rate of 3 - 4 ml / s). After the injection of the contrast agent, double helix scan in the arterial phase and portal venous phase were acquired. In order to avoid the possibility of image information loss, we obtained DICOM images directly from the picture archiving and communication system (PACS) system without compression and down sampling.

### Radiomic Analysis

**Figure 1:**
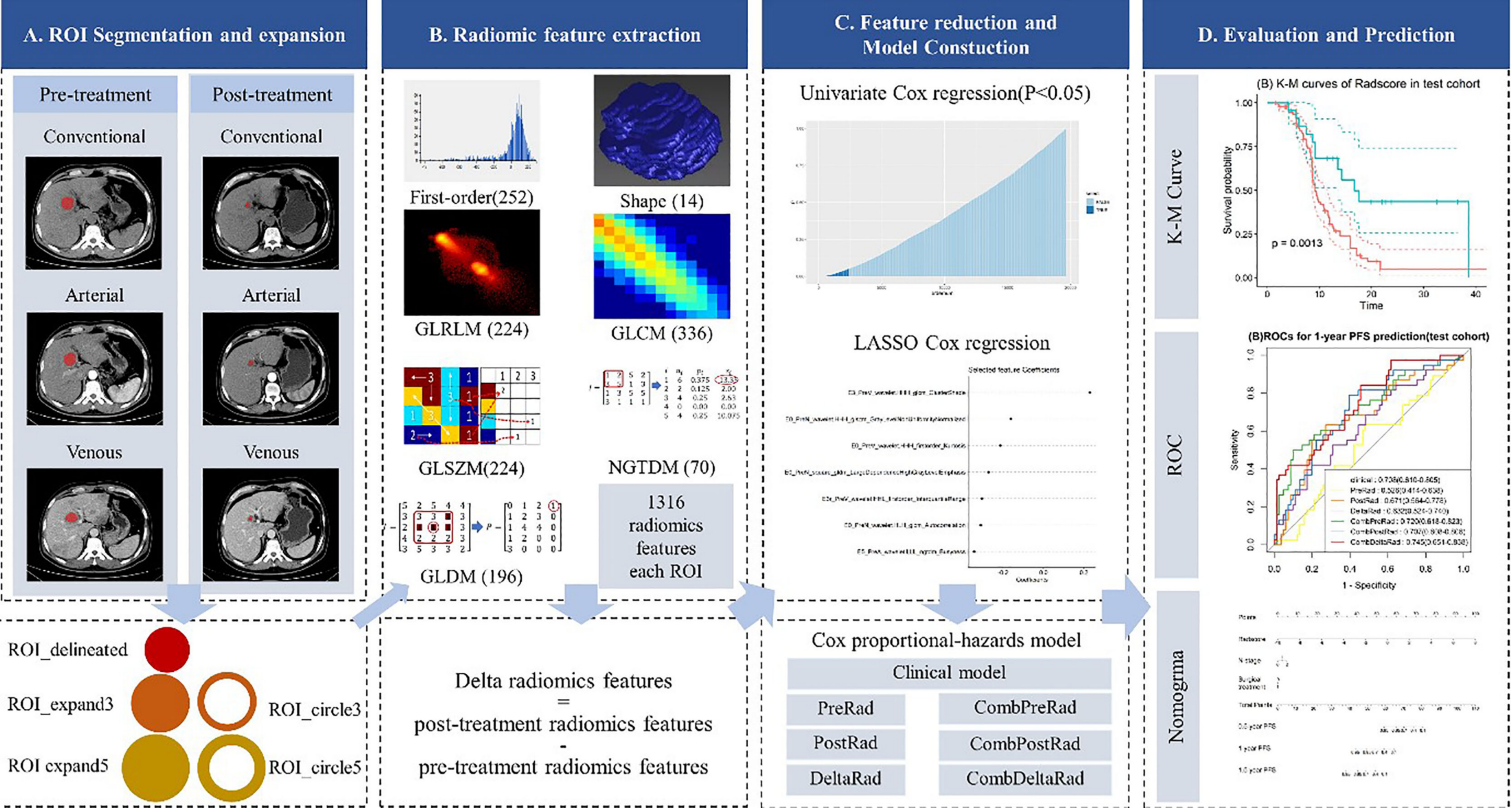
Radiomics framework of predicting the PFS of patients with colorectal liver metastases undergo neoadjuvant chemotherapy. PFS, progress-free survival.

The four steps of radiomic analysis workflow are presented in **Figure 1**, including lesion segmentation, image preprocessing, radiomic feature extraction, radiomic feature selection, model building, and model evaluation and application.

### Lesion Segmentation

Two-dimensional manual segmentation of lesion in axial image of plain scanning, arterial, and venous phases was performed by an open-source ITK-SNAP software (www.itksnap.org). The largest section of the lesion was selected. Regions-of-interest (ROI) were manually delineated by a radiologist who had 5 years of experience in abdomen CT imaging interpretation and checked by a senior radiologist with 5 years of experience in liver CT imaging interpretation. Because of the presence of patients with multiple metastases, only the largest five lesions were selected for analysis in each patient. When the number of metastases was greater than 5, we chose to include all of them.

### Image Preprocessing and Radiomic Feature Extraction

Image preprocessing and radiomic feature extraction were carried out with in-house software (Artificial Intelligence Kit, v. 3.3.0, GE Healthcare). The steps are as follows: firstly, every image and the corresponding ROI were resampled to a uniform pixel dimension size of 1 × 1 mm^2^; and secondly, each delineated ROI (ROI_delineated) was expanded the edge by 3mm and 5mm in AK software, automatically. In this way, we acquired 2 circular ROIs (ROI_circle3 and ROI_circle5) and two enlarged ROIs (ROI_expand3 and ROI expand5). The diagram of ROI expansion is shown in **Figure 1**. Each circular ROI and enlarged ROI were checked. The ROI was manually corrected for regions outside of liver parenchyma. Finally, we performed radiomics feature extraction on ROI_delineated, ROI_circle3, ROI_circle5, ROI_ expand3 and ROI_expand5. For each phase (non-enhanced, arteria,l and venous images phase) in pre-treatment and post-treatment CT images, we extract 6,580 radiomic features (1,316 radiomic features for one ROI, with 5 ROI in total). Finally, we obtained 19,740 features on the pre-treatment and post-treatment CT images, respectively. In order to explore the changes in the feature values before and after the treatment, we obtained the delta feature by subtracting the features after the treatment from the features before the treatment.

Among these 1,316 features, 7 categories of features were extracted: shape features (n = 14), first order features (n = 252), Gray Level Co-occurrence Matrix (GLCM, n = 336), Gray Level Run Length Matrix (GLRLM, n = 224), Gray Level Size Zone Matrix (GLSZM, n = 224), Gray Level Dependence Matrix (GLDM, n = 196), Neighboring Gray Tone Difference Matrix (NGTDM, n = 70).

### Radiomic Feature Selection

Patients were randomly assigned to training or testing groups and we ensured that the ratio of the number of lesions in the training and testing groups was 7:3. All cases in the training cohort were used to train the predictive model and cases in the testing cohorts were used to evaluate the model’s performance. Features with zero variance were excluded and missing values and outliers were replaced by the median. Finally, the Z-score is used to standardize the data and the data of different magnitudes are uniformly converted into the same magnitude to ensure the comparability between the data (25, 26).

Although a large number of radiomics features were extracted in previous step, not all of them are relevant to the prognosis of patients with liver metastases from colorectal cancer. In this study, the univariate Cox regression and least absolute shrinkage and selection operator (LASSO) Cox regression were used to select valuable features from the training cohort. Univariate Cox proportional hazard regression model was first applied to each feature. If the p-value of a feature in the univariate Cox model was less than 0.05, the feature was selected, otherwise the feature was removed. LASSO Cox regression was then performed for multivariate feature selection by introducing a penalizing parameter (lambda). Tuning lambda can affect the weight coefficients of each feature and the performance of the Lasso cox model. The features with a weight coefficient of 0 in Lasso cox model were eliminated. In order to get an optimal feature number and avoid over-fitting, the parameter tuning was performed under ten-fold cross-validation. The parameters were finally determined by the performance of LASSO Cox model.

### Model Building

Multivariate Cox proportional hazard model was used to build a prognostic prediction model for liver metastasis from colorectal cancer.

#### Clinical Model

Seven Candidate clinical variables for the prognosis of liver metastasis from colorectal cancer were selected, including age, sex, T stage, N stage, M stage, metastasis status, and surgical treatment. Univariate Cox regression was used to assess predictive power of the clinical candidates. Clinical variables with a P value less than 0.05 were selected to construct clinical models.

#### Radiomics Model

Three radiomic models were constructed using radiomic features extracted from pre-treatment, post-treatment images, and differences between the two, namely pre-treatment radiomics model (PreRad), post-treatment radiomics model (PostRad), and delta radiomics model (DeltaRad). The feature selection process is shown in section “Radiomic Feature selection”. A radiomics score (Rad-Score) was calculated for each patient by linear combination of radiomic features with associated weights.

#### Combined Model

Radscores from pre- and post-treatment imaging features and their differences were separately combined with clinical features significant in the univariate analysis to form three combined prediction models: CombPreRad, CombPostRad, and CombDeltaRad.

### Model Evaluation and Application

C-index was used to evaluate model performance. It reflects the consistency between the PFS predicted by the model and the actual PFS of all patients. The value range of C-index is 0.5-1 A C-index of 0.5 indicates that the predicted value is poor and a C-index greater than 0.7 indicates moderate to excellent performance.

Based on result of the three models in the training cohort, X-tile was performed to stratify patients into high-risk group and low-risk group in both training and testing cohorts. The Kaplan–Meier (KM) survival curve analyses of PFS was performed and log-tank test is used to compare the difference in survival curves between high- and low- risk groups.

In addition, we evaluated the effectiveness of the combined COX model in predicting the probability of PFS at a given time point (PFS of 1 years in this study). Receiver operating characteristic (ROC) analysis was performed to estimate the prognostic performance of the three combined models in predicting 1-year PFS. The calibration curves and Hosmer-Lemeshow test were utilized to assess the agreement between predicted and actual probabilities of various models. The net reclassification index (NRI) and total integrated discrimination index (IDI) were used to assess the clinical benefit of different models.

For model visualization and clinical application, we constructed a nomogram based on the model with the highest discriminative efficiency.

### Statistical Analysis

All statistical analyses were performed using R 3.6.1. Where appropriate, the two-sample t-test, chi-square test, or Mann-Whitney U test was applied to the training and validation cohorts to assess clinical findings, image characteristics, and median PFS time. The Lasso-based feature selection, C-index calculation, and X-tile-based threshold acquisition were implemented using the “glmnet”, “survcomp” and “survminer” package, respectively. The Cox proportional hazard model construction, Kaplan-Meier curve analysis, and Log-rank test used the “survival “ package. The construction of nomogram and calibration curve were implemented with the “rms” package. A two-tailed P-value less than 0.05 was considered statistically significant.

## Result

### Patient Characteristics

In this study, 397 lesions from 139 patients were included. The patient characteristics are presented in **Table 1**. The clinical characteristics of different lesions in the same patient are assigned the same value as the patient. The average age of the patients was 56.96 ± 11.06 years. The number of male patients was 96 (69.06%). There were 43 (30.94%), 22 (15.83%), 22 (15.83%), 15 (10.79%) and 37 (26.62%) patients with 1, 2, 3, 4 and 5 metastases lesions, respectively. The mean PFS time was 11.80 ± 7.93 months.

**Table 1:** Clinical characteristics of patients.

Characteristics	Patients (N = 139)
**Age (years, mean ± SD)**	56.96 ± 11.06
**Gender (%)**	
Male	96 (69.06%)
Female	43 (30.94%)
**cT stage (%)**	
3	85 (61.15%)
4	54 (38.85%)
**cM.stage (%)**	
**1**	139 (100.00%)
**cN stage (%)**	
0	6 (4.32%)
1	36 (25.90%)
2	97 (69.78%)
**Metastasis to other sites**	
No	93 (66.91%)
Yes	46 (33.09%)
**Surgical treatment**	
No	78 (56.12%)
Yes	61 (43.88%)
**Number of metastatic lesions per patient**	
1	43 (30.94%)
2	22 (15.83%)
3	22 (15.83%)
4	15 (10.79%)
5	37 (26.62%)
**Mean PFS time (months, mean ± SD)**	11.80 ± 7.93

### Feature Selection and Radiomics Signature Construction

Ct-Stage and surgical treatment were significant clinical features in the univariate Cox regression analysis. The HR values and 95% confidence interval (CI) of Ct-stage and surgical treatment in multivariate cox regression are 2.033 (1.507~0.433) and 0.582 (1.507~0.433), respectively. These two clinical features were used to construct a clinical model.

Based on the dimensionality reduction methods of univariate cox regression and lasso cox regression analysis, the PreRad, PostRad and DeltaRad models are constructed by 7, 15, and 24 features, respectively.

**Figure 2:**
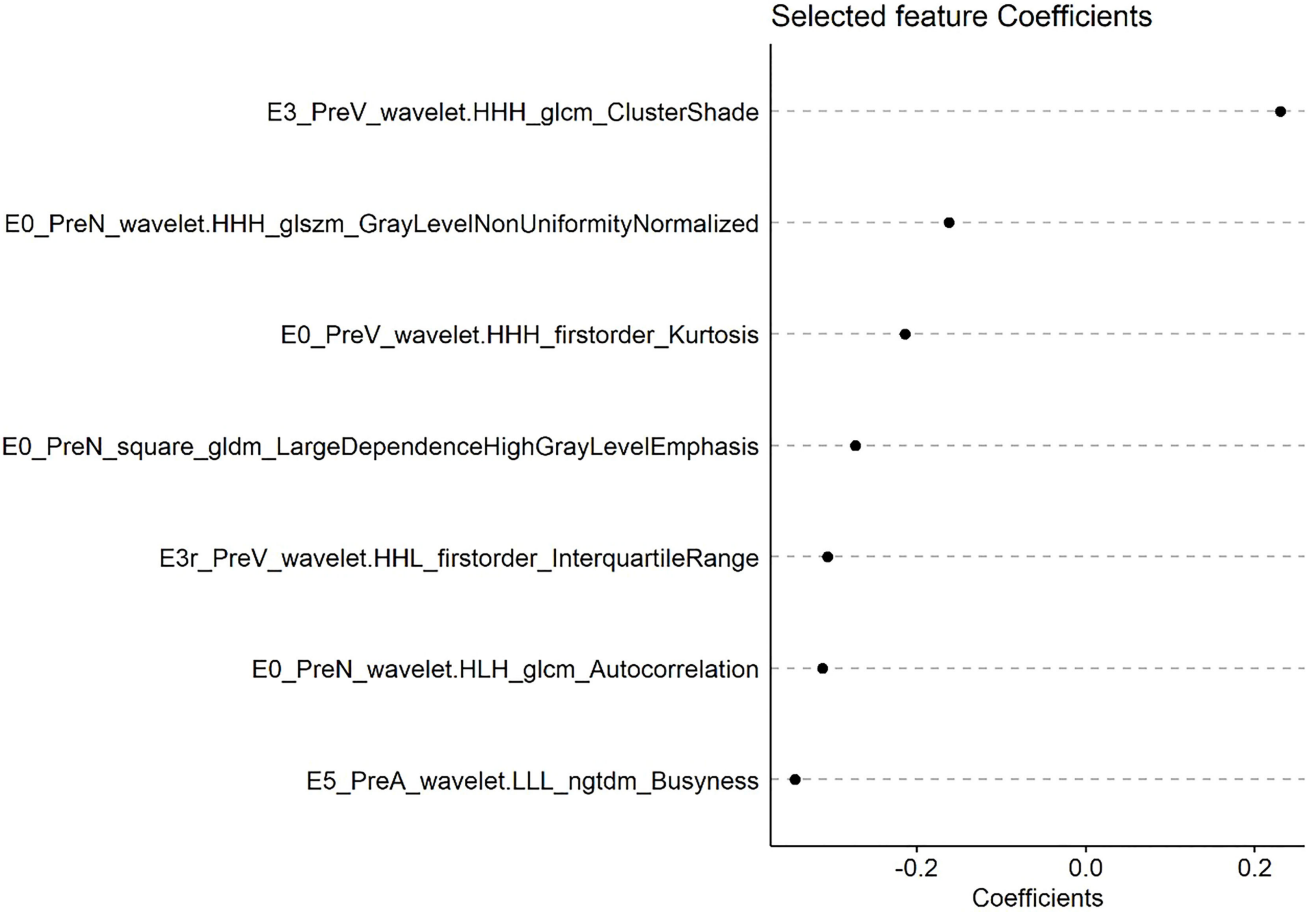
The coefficients of each feature based on PreRad model.

A radscore was calculated based on radiomic features and their associated weight from lasso cox model. **Figure 2** shows the coefficients of each feature based on PreRad model.

### PFS Prediction Performance of Various Models

Seven models were constructed by combining clinical variable with radscore calculated from CT images before and after treatment, namely clinical, namely PreRad, PostRad, DeltaRad, CombPreRad, CombPostRad, and CombDeltaRad, respectively. The C-index of these models in the training cohort and testing cohort are shown in **Table 2**.

**Table 2:** PFS prediction performance of various models.

Models	Training cohort C-index (95 CI %)	Testing cohort C-index (95 CI %)
Clinical	0.661 (0.600-0.721)	0.673 (0.583-0.763)
PreRad	0.669 (0.626-0.712)	0.614 (0.552-0.675)
PostRad	0.757 (0.721-0.793)	0.642 (0.578-0.707)
DeltaRad	0.800 (0.771-0.829)	0.688 (0.627-0.749)
CombPreRad	0.701 (0.662-0.740)	0.696 (0.638-0.754)
CombPostRad	0.763 (0.728-0.798)	0.694 (0.633-0.755)
CombDeltaRad	0.802 (0.772-0.832)	0.744 (0.686-0.803)

The performance of the clinical model is moderate, with a C-index value and 95% CI of 0.661(0.600-0.721) and 0.673 (0.583-0.763) in the training and testing cohort. The combined model demonstrated an increased performance. For radiomics features, the performance is ranked as DeltaRad, PostRad, and PreRad in descending order, both in training and testing cohort. CombDeltaRad achieved the best performance in both training cohort (C-index (95% CI): 0.802(0.772-0.832)) and the testing cohort (0.744(0.686-0.803)).

### 1-Year PFS Probability Prediction of Various Models

**Figure 3:**
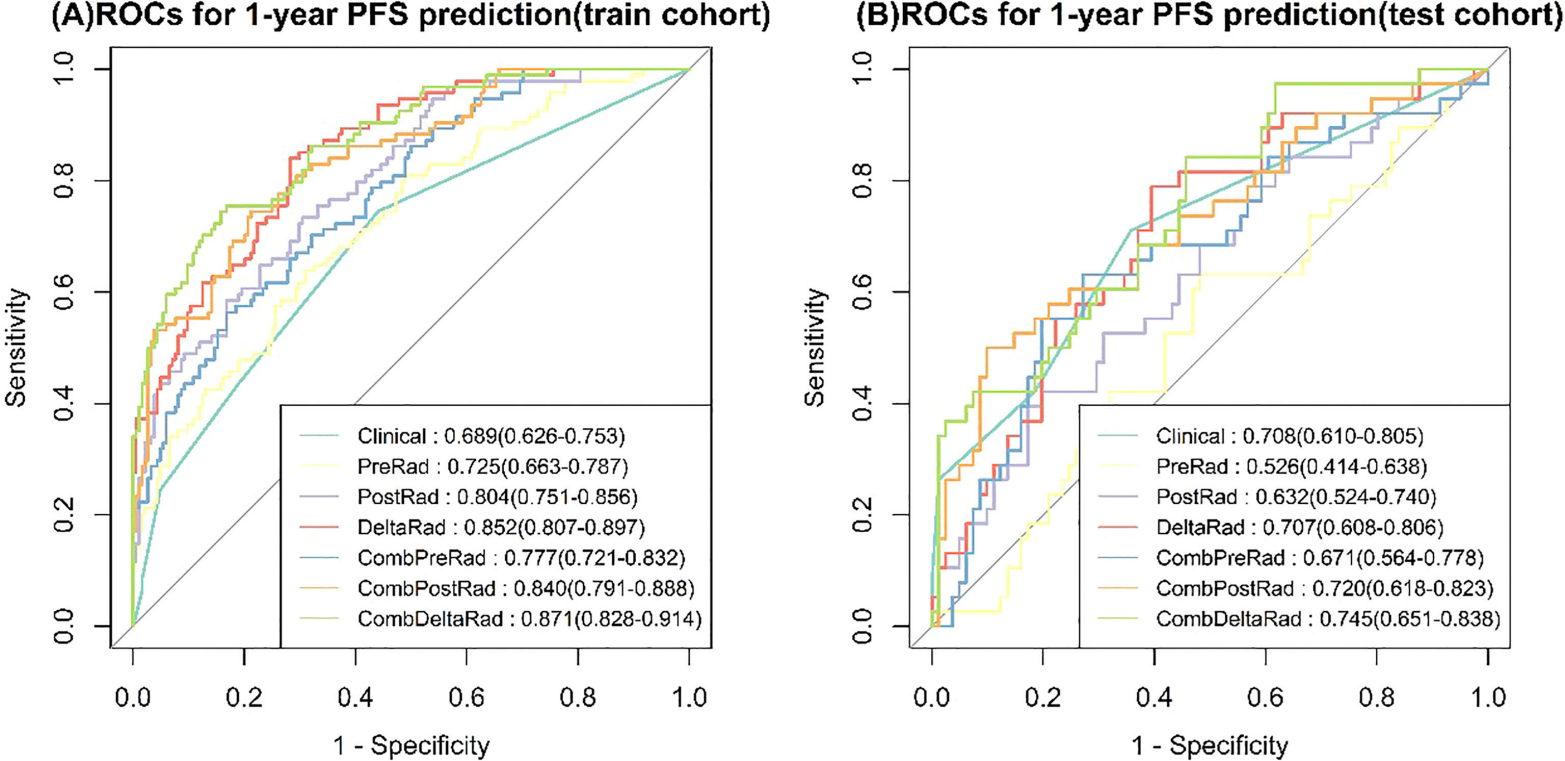
ROCs for 1-year PFS probability prediction of various Models. **(A)** train cohort, **(B)** test cohort.

For 1-year PFS probability prediction, the training and testing performance of different models are shown in **Table 3**. The result is consistent with PFS prediction performance. CombDeltaRad model obtained the best performance with area under the curve (AUC) and 95% CI of 0.871(0.828-0.914) and 0.745(0.651-0.838) in training cohort and testing cohort, respectively. The ROCs for 1-year PFS probability prediction of various models are presented in **Figure 3**. The calibration curves and Hosmer-Lemeshow test results of various models are presented in **Supplementary Material**. The p-value of both the training cohort and the testing cohort of the CombDeltaRad model is greater than 0.05. The reclassification measures of discrimination confirmed that DeltaRad, CombPreRad, CombPostRad and CombDeltaRad better than the clinical models with NRI of 0.042 [-0.205 - 0.289], 0.008 [-0.180 - 0.195], 0.049 [-0.135 - 0.232] and 0.033 [-0.188 - 0.254] Respectively; and IDI of 0.034 [-0.166 - 0.233], 0.006 [-0.146 - 0.158], 0.039 [-0.109 - 0.187] and 0.026 [-0.152 - 0.205], respectively (**Supplementary Material**, **Supplementary Tables 2, 3**).

**Table 3:** 1-year PFS prediction performance of various models.

Models	cohort	AUC (95% CI)	ACC (95% CI)	Sensitivity (95% CI)	Specificity (95% CI)
Clinical	training	0.689 (0.626-0.753)	0.622 (0.621-0.624)	0.745 (0.657-0.833)	0.560 (0.488-0.632)
	testing	0.708 (0.610-0.805)	0.664 (0.660-0.668)	0.711 (0.566-0.855)	0.642 (0.538-0.746)
PreRad	training	0.725 (0.663-0.787)	0.673 (0.671-0.674)	0.638 (0.541-0.735)	0.690 (0.623-0.757)
	testing	0.526 (0.414-0.638)	0.395 (0.391-0.399)	0.974 (0.923-1.025)	0.123 (0.052-0.195)
PostRad	training	0.804 (0.751-0.856)	0.709 (0.707-0.710)	0.734 (0.645-0.823)	0.696 (0.629-0.762)
	testing	0.632 (0.524-0.740)	0.681 (0.677-0.684)	0.421 (0.264-0.578)	0.802 (0.716-0.889)
DeltaRad	training	0.852 (0.807-0.897)	0.759 (0.758-0.760)	0.840 (0.766-0.914)	0.717 (0.652-0.782)
	testing	0.707 (0.608-0.806)	0.664 (0.660-0.668)	0.789 (0.660-0.919)	0.605 (0.498-0.711)
CombPreRad	training	0.777 (0.721-0.832)	0.741 (0.740-0.742)	0.564 (0.464-0.664)	0.832 (0.777-0.886)
	testing	0.671 (0.564-0.778)	0.697 (0.694-0.701)	0.632 (0.478-0.785)	0.728 (0.632-0.825)
CombPostRad	training	0.840 (0.791-0.888)	0.773 (0.772-0.775)	0.745 (0.657-0.833)	0.788 (0.729-0.847)
	testing	0.720 (0.618-0.823)	0.773 (0.770-0.776)	0.500 (0.341-0.659)	0.901 (0.836-0.966)
CombDeltaRad	training	0.871 (0.828-0.914)	0.809 (0.808-0.810)	0.745 (0.657-0.833)	0.842 (0.790-0.895)
	testing	0.745 (0.651-0.838)	0.639 (0.635-0.642)	0.842 (0.726-0.958)	0.543 (0.435-0.652)

### Kaplan-Meier Analysis

**Figure 4:**
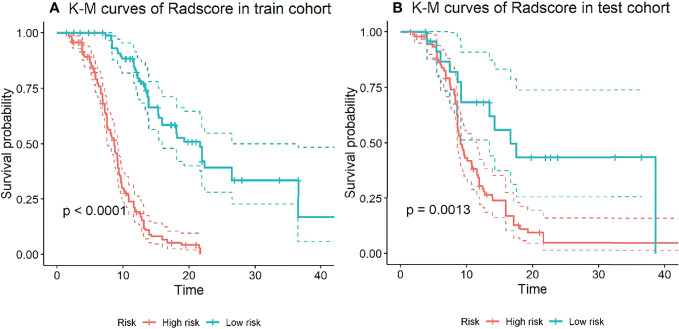
Kaplan-Meier analysis of PFS based on CombDeltaRad model. **(A)** train cohort, **(B)** test cohort.

The X-tile method was used to determine the cutoff value of the CombDeltaRad model in training cohort data. Then, the patients were divided into high-risk groups and low-risk groups based on this cutoff value. In this study, the cutoff value of the CombDeltaRad model was 0.183. **Figure 4** shows the Kaplan-Meier analysis of the CombDeltaRad model.

For the training cohort, the median PFS times were 8.67 months and 21.67 months in the high-risk and low-risk groups, respectively. For the testing cohort, the median PFS times were 9.17 months and 16.70 months in the high-risk and low-risk groups, respectively. There were significant differences between the low- and high-risk groups (log-rank test, P < 0.0001, P = 0.0013, respectively).

### Nomogram Construction

**Figure 5:**
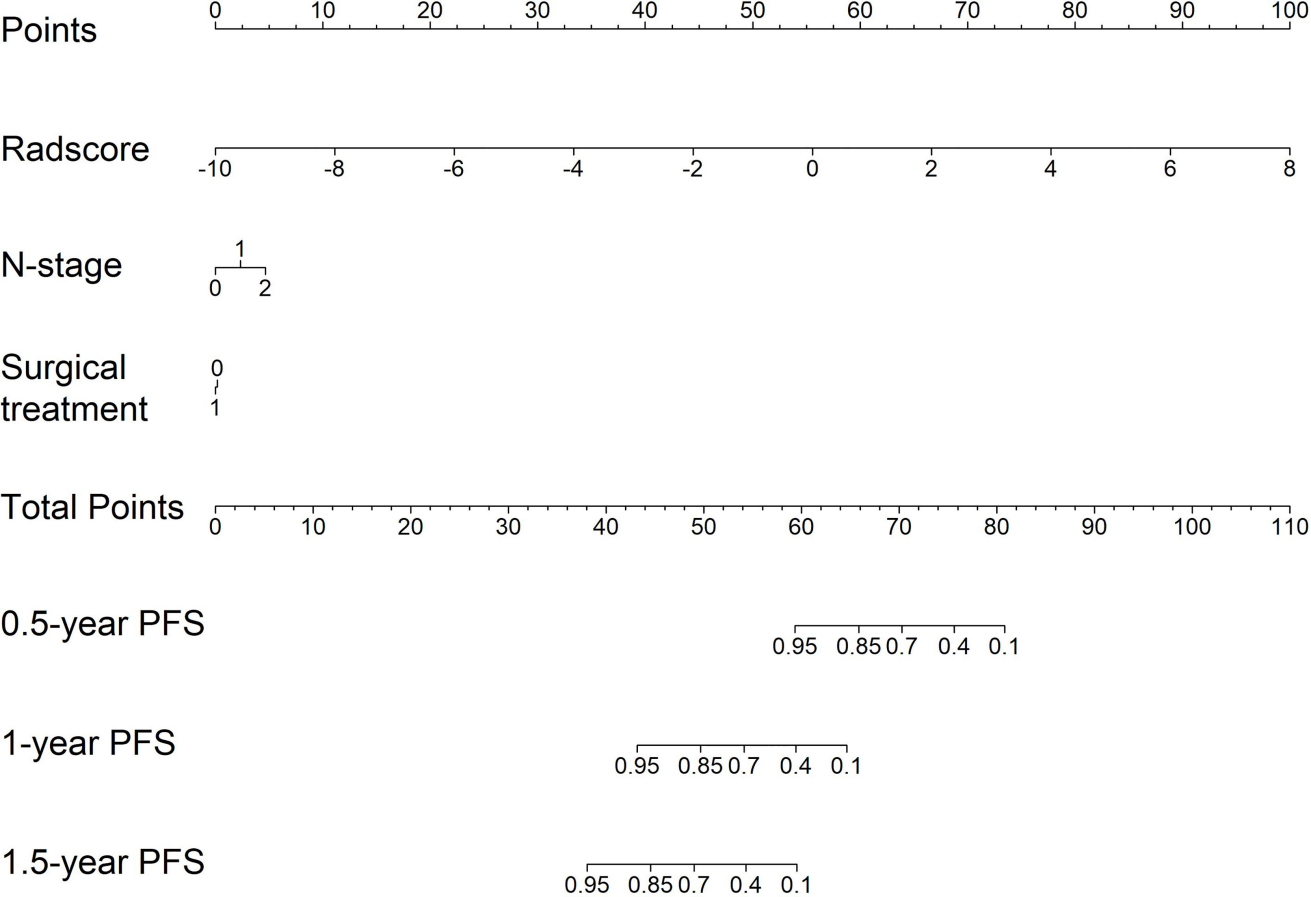
The nomogram for PFS probability prediction based on CombDeltaRad model.

For clinical use, we built a nomogram based on the CombDeltaRad model (**Figure 5**). The nomogram consists of three factors: N-stage, surgical treatment, and radscore (delta). A total score was calculated by summing the scores of each factor for each patient. The higher the score, the lower the 1-, 3-, and 5- year survival probability.

## Discussion

Previous studies have used a large number of clinical, pathological, and molecular factors to predict the survival rate of patients with colorectal cancer after hepatectomy (27–33). This includes: the stage of primary tumor, preoperative serum CEA concentration, the size and number of metastases, whether there is extrahepatic metastasis, or the size of the retained edge during resection. Based on these data, people have established the survival scale, of which the most widely recognized is described by Fong et al. and Iwatsuki et al. (34, 35). However, these studies rarely extract useful texture features and clinical features through imaging images. At the same time, it is difficult to predict the curative effect for the lesions with no obvious imaging changes in a short time. In order to solve this problem, in our study, we predicted the efficacy and prognostic value of CT imaging features in patients with liver metastasis after neoadjuvant chemotherapy. The results show that this method has a good prediction effect on PFS. Our research will help to predict the efficacy and progress of patients after neoadjuvant chemotherapy through image texture features and changes, so as to assist in the clinical treatment decision-making process.

Image feature extraction includes conventional scanning, arterial scanning, and venous scanning, which helps to increase the number of extracted features adding diversity of feature extraction. We chose 2D ROI for sketching, because while previous studies have shown that both 3D and 2D segmentation are reliable, the 2D method is more practical, time-saving, and can reduce the contour change between readings (34).

When clinical features were combined with radiomics features, CombDeltaRad performed best in the training cohort (Cindex (95% CI): 0.802 (0.772-0.832)) and the testing cohort Cinde (95% CI): 0.744 (0.686-0.803)); The AUC (95% CI) of CombDeltaRad model obtained the best performance in the training queue 0.871 (0.828-0.914) and the test queue 0.745 (0.651-0.838). The one-year PFS prediction was consistent with the PFS prediction performance. This shows that when combining clinical features with radiological features, the radiological score can provide more prognostic information than a single clinical or radiological feature through clinical features and extracted image features. Therefore, it can be used as a reference to obtain prognosis, so as to provide the ability to improve or predict prognosis. At the same time, radscore can divide patients into high-risk group and low-risk groups, which is helpful to the stratification of patients. Patients with higher radscore have poorer PFS, suggesting that the risk of recurrence and metastasis is higher, and early treatment is more favorable. Our findings will open a key step to enable surgeons to tailor different treatment options for high-risk and low-risk colorectal cancer patients with liver metastasis according to specific clinical and radiological characteristics.

Among the 1,316 radiological features, 7 categories of image features were extracted and combined with clinical features to obtain 3 combined models, namely CombPreRad, CombPostRad, and CombDeltaRad. Finally, 24 were identified as predictive features of PFS, of which 16 were wavelet features, which may indicate that wavelet features contain more prognostic information. In addition, two logarithmic features are extracted from the image. Wavelet features reflect tumor information from eight spatial domains and logarithmic features reflect tumor information from three frequency domains. This result shows that much prediction information can be mined by wavelet and logarithmic transformation of the original image. This further reflects the advantages of the radiomics method, because it is good at mining high-dimensional information which is difficult to perceive manually. For example, selecting “SumEntropy” in wavelet subspace and “skewness” in logarithmic subspace shows that tumor heterogeneity described by entropy and tumor intensity has prognostic value in high-dimensional wavelet and logarithmic space.

Although the radiology score was good, our study had some limitations. Firstly, we mine seven features from CT images and combine their manifestations with clinical factors. However, the association between radioactive characteristics and biological level events has not been explained. Secondly, this study is a single center study and the sample size is small. In the future, we plan to cooperate with multiple centers for research and plan to combine genotyping with radiation characteristics. Thirdly, the longest PFS of the data we included is only 43 months and the current model may not be able to predict long-term PFS. We will continue to follow up the data and verify the model’s prediction of long-term PFS in the next step. In addition, as an emerging method in medical image analysis, deep learning can provide valuable features and supplement radiological features (35).

In conclusion, this study provides a noninvasive and preprocessing method for CT based PFS of colorectal cancer liver metastasis. In addition, for each patient with colorectal cancer liver metastasis, the radiation score can divide the patients into a high-risk group and a low-risk group. This study may provide some important insights for precise treatment and valuable guidance for clinicians.

## Data Availability Statement

The original contributions presented in the study are included in the article/**Supplementary Material**. Further inquiries can be directed to the corresponding author.

## Author Contributions

SY, YH and XP are co-authors. SY: Formal analysis, Investigation, Writing - original draft. YH: Investigation KN: Investigation YL: Methodology, Software. XCM: Conceptualization, Supervision, writing – review and editing. XP participated in data collection, data analyses, software, and interpretation. All authors contributed to the article and approved the submitted version.

## Funding

This study was supported by grants from the Guangdong Natural Science Foundation (2021A1515011795).

## Conflict of Interest

Author YTL was employed by GE Healthcare Pharmaceutical Diagnostics.

The remaining authors declare that the research was conducted in the absence of any commercial or financial relationships that could be construed as a potential conflict of interest.

## Publisher’s Note

All claims expressed in this article are solely those of the authors and do not necessarily represent those of their affiliated organizations, or those of the publisher, the editors and the reviewers. Any product that may be evaluated in this article, or claim that may be made by its manufacturer, is not guaranteed or endorsed by the publisher.

## References

[1] Sung H Ferlay J Siegel RL Laversanne M Soerjomataram I Jemal A. Global Cancer Statistics 2020: GLOBOCAN Estimates of Incidence and Mortality Worldwide for 36 Cancers in 185 Countries. CA Cancer J Clin (2021) 71(3):209–49. doi: 10.3322/caac.21660 33538338

[2] Lee WS Yun SH Chun HK Lee WY Yun HR Kim J. Pulmonary Resection for Metastases From Colorectal Cancer: Prognostic Factors and Survival. Int J Colorect Dis (2007) 22:699–704. doi: 10.1007/s00384-006-0218-2 17109105

[3] Van Cutsem E Nordlinger B Adam R Köhne CH Pozzo C Poston G. Towards a Paneuropean Consensus on the Treatment of Patients With Colorectal Liver Metastases. Eur J Cancer (2006) 42:2212–21. doi: 10.1016/j.ejca.2006.04.012 16904315

[4] Yoo PS Lopez-Soler RI Longo WE Cha CH. Liver Resection for Metastatic Colorectal Cancer in the Age of Neoadjuvant Chemotherapy and Bevacizumab. Clin Colorectal Cancer (2006) 6:202–7. doi: 10.3816/CCC.2006.n.036 17026789

[5] Fong Y Cohen AM Fortner JG Enker WE Turnbull AD Coit DG. Liver Resection for Colorectal Metastases. J Clin Oncol (1997) 15:938–46. doi: 10.1200/JCO.1997.15.3.938 9060531

[6] Muratore A Zorzi D Bouzari H Amisano M Massucco P Sperti E. Asymptomatic Colorectal Cancer With Un-Resectable Liver Metastases: Immediate Colorectal Resection or Up-Front Systemic Chemotherapy? Ann Surg Oncol (2007) 14:766–70. doi: 10.1245/s10434-006-9146-1 17103261

[7] Hayashi M Inoue Y Komeda K Shimizu T Asakuma M Hirokawa F. Clinicopathological Analysis of Recurrence Patterns and Prognostic Factors for Survival After Hepatectomy for Colorectal Liver Metastasis. BMC Surg (2010) 10:27. doi: 10.1186/1471-2482-10-27 20875094 PMC2949597

[8] Manfredi S Lepage C Hatem C Coatmeur O Faivre J Bouvier AM. Epidemiology and Management of Liver Metastases From Colorectal Cancer. Ann Surg (2006) 244(2):254–9. doi: 10.1097/01.sla.0000217629.94941.cf PMC1602156 16858188

[9] Noren A Eriksson HG Olsson LI. Selection for Surgery and Survival of Synchronous Colorectal Liver Metastases; a Nationwide Study. Eur J Cancer (2016) 53:105–14. doi: 10.1016/j.ejca.2015.10.055 26702764

[10] Bipat S Niekel MC Comans EF Nio CY Bemelman WA Verhoef C. Imaging Modalities for the Staging of Patients With Colorectal Cancer. Netherl J Med (2012) 70(1):26–34. doi: 10.3402/ljm.v7i0.19086 22271811

[11] Alberts SR Horvath WL Sternfeld WC Goldberg RM Mahoney MR Dakhil SR. Oxaliplatin, Fluorouracil, and Leucovorin for Patients With Unresectable Liver-Only Metastases From Colorectal Cancer: A North Central Cancer Treatment Group Phase II Study. J Clin Oncol (2005) 23:9243–9. doi: 10.1200/JCO.2005.07.740 16230673

[12] Dawood O Mahadevan A Goodman KA. Stereotactic Body Radiation Therapy for Liver Metastases. Eur J Cancer (2009) 45:29472959. doi: 10.1016/j.ejca.2009.08.011 19773153

[13] Kemeny N. Management of Liver Metastases From Colorectal Cancer. Oncol (Williston Park) (2006) 20(10):1161–76, 1179; discussion 1179–80, 1185–6. 17024869

[14] Eisenhauer E Therasse P Bogaerts J Schwartz L Sargent D Ford R. New Response Evaluation Criteria in Solid Tumours: Revised RECIST Guideline (Version 1.1). Eur J Cancer (2009) 45(2):228–47. doi: 10.1016/j.ejca.2008.10.026 19097774

[15] Robinson PJ. The Effects of Cancer Chemotherapy on Liver Imaging. Eur Radiol (2009) 19(7):1752–62. doi: 10.1007/s00330-009-1333-6 19238392

[16] Therasse P Arbuck SG Eisenhauer EA Wanders J Kaplan RS Rubinstein L. New Guidelines to Evaluate the Response to Treatment in Solid Tumors. European Organization for Research and Treatment of Cancer, National Cancer Institute of the United States, National Cancer Institute of Canada. J Natl Cancer Inst (2000) 92(3):205–16. doi: 10.1093/jnci/92.3.205 10655437

[17] Castellano G Bonilha L Li LM Cendes F. Texture Analysis of Medical Images. Clin Radiol (2004) 59(12):1061–9. doi: 10.1016/j.crad.2004.07.008 15556588

[18] Niekel MC Bipat S Stoker J. Diagnostic Imaging of Colorectal Liver Metastases With CT, MR Imaging, FDG PET, and/or FDG PET/CT: A Meta-Analysis of Prospective Studies Including Patients Who Have Not Previously Undergone Treatment. Radiology (2010) 257(3):674–84. doi: 10.1148/radiol.10100729 20829538

[19] Lubner MG Stabo N Lubner SJ del Rio AM Song C Halberg RB. CT Textural Analysis of Hepatic Metastatic Colorectal Cancer: Pre-Treatment Tumor Heterogeneity Correlates With Pathology and Clinical Outcomes. Abdom Imaging (2015) 40(7):2331–7. doi: 10.1007/s00261-015-0438-4 25968046

[20] Miles KA Ganeshan B Griffiths MR Young RC Chatwin CR. Colorectal Cancer: Texture Analysis of Portal Phase Hepatic CT Images as a Potential Marker of Survival. Radiology (2009) 250(2):444–52. doi: 10.1148/radiol.2502071879 19164695

[21] Rao SX Lambregts DM Schnerr RS Beckers RC Maas M Albarello F. CT Texture Analysis in Colorectal Liver Metastases: A Better Way Than Size and Volume Measurements to Assess Response to Chemotherapy? United Eur Gastroenterol J (2016) 4(2):257–63. doi: 10.1177/2050640615601603 PMC4804371 27087955

[22] Caruso D Zerunian M Ciolina M de Santis D Rengo M Soomro MH. Haralick’s Texture Features for the Prediction of Response to Therapy in Colorectal Cancer: A Preliminary Study. Radiol Med (2017) 123(3):161–7. doi: 10.1007/s11547-017-0833-8 29119525

[23] Beckers RCJ Lambregts DMJ Schnerr RS Maas M Rao S-X Kessels AGH. Whole Liver CT Texture Analysis to Predict the Development of Colorectal Liver Metastases—A Multicentre Study. Eur J Radiol (2017) 92:64–71. doi: 10.1016/j.ejrad.2017.04.019 28624022

[24] Rao SX Lambregts DM Schnerr RS van Ommen W van Nijnatten TJ Martens MH. Whole-Liver CT Texture Analysis in Colorectal Cancer: Does the Presence of Liver Metastases Affect the Texture of the Remaining Liver? United Eur Gastroenterol J (2014) 2(6):530–8. doi: 10.1177/2050640614552463 PMC4245301 25452849

[25] Ko J Park U Kim D Kang SW. Quantitative Electroencephalogram Standardization: A Sex- and Age-Differentiated Normative Database. Front Neurosci (2021) 15:766781. doi: 10.3389/fnins.2021.766781 34975376 PMC8718919

[26] Haga A Takahashi W Aoki S Nawa K Yamashita H Abe O. Standardization of Imaging Features for Radiomics Analysis. J Med Invest (2019) 66(1.2):35–7. doi: 10.2152/jmi.66.35 31064950

[27] Fong Y Fortner J Sun RL Brennan MF Blumgart LH. Clinical Score for Predicting Recurrence After Hepatic Resection for Metastatic Colorectal Cancer: Analysis of 1001 Consecutive Cases. Ann Surg (1999) 230:309–18. doi: 10.1097/00000658-199909000-00004 PMC1420876 10493478

[28] Iwatsuki S Dvorchik I Madariaga JR Marsh JW Dodson F Bonham AC. Hepatic Resection for Metastatic Colorectal Adenocarcinoma: A Proposal of a Prognostic Scoring System. J Am Coll Surg (1999) 189:291–99. doi: 10.1016/S1072-7515(99)00089-7 PMC2967754 10472930

[29] Mann CD Metcalfe MS Leopardi LN Maddern GJ. The Clinical Risk Score: Emerging as a Reliable Preoperative Prognostic Index in Hepatectomy for Colorectal Metastases. Arch Surg (2004) 139:1168–72. doi: 10.1001/archsurg.139.11.1168 15545561

[30] Rees M Tekkis PP Welsh FK O'Rourke T John TG. Evaluation of Long-Term Survival After Hepatic Resection for Metastatic Colorectal Cancer: A Multifactorial Model of 929 Patients. Ann Surg (2008) 247:125–35. doi: 10.1097/SLA.0b013e31815aa2c2 18156932

[31] John SK Robinson SM Rehman S Harrison B Vallance A French JJ. Prognostic Factors and Survival After Resection of Colorectal Liver Metastasis in the Era of Preoperative Chemotherapy: An 11-Year Single-Centre Study. Dig Surg (2013) 30:293–301. doi: 10.1159/000354310 23969407

[32] Nash GM Gimbel M Shia J Nathanson DR Ndubuisi MI Zeng ZS. KRAS Mutation Correlates With Accelerated Metastatic Progression in Patients With Colorectal Liver Metastases. Ann Surg Oncol (2010) 17:572–78. doi: 10.1245/s10434-009-0605-3 19727962

[33] Smith DL Soria JC Morat L Yang Q Sabatier L Liu DD. Human Telomerase Reverse Transcriptase (hTERT) an Ki-67 Are Better Predictors of Survival Than Established Clinical Indicators in Patients Undergoing Curative Hepatic Resection for Colorectal Metastases. Ann Surg Oncol (2004) 11:45–51. doi: 10.1007/BF02524345 14699033

[34] Rizzetto F Calderoni F De Mattia C Defeudis A Giannini V Mazzetti S. Impact of Inter-Reader Contouring Variability on Textural Radiomics of Colorectal Liver Metastases. Eur Radiol Exp (2020) 4(1):62. doi: 10.1186/s41747-020-00189-8 33169295 PMC7652946

[35] Li H Xu C Xin B Zheng C Zhao Y Hao K. 18f-FDG PET/CT Radiomic Analysis With Machine Learning for Identifying Bone Marrow Involvement in the Patients With Suspected Relapsed Acute Leukemia. Theranostics (2019) 9(16):4730–9. doi: 10.7150/thno.33841 PMC6643435 31367253

