# Corrigendum: Association of CT-based delta radiomics biomarker with progression-free survival in patients with colorectal liver metastases undergo chemotherapy

**DOI:** 10.3389/fonc.2023.1283480

**Published:** 2023-09-18

**Authors:** Shuai Ye, Yu Han, XiMin Pan, KeXin Niu, YuTing Liao, XiaoChun Meng

**Affiliations:** ^1^ The Sixth Affiliated Hospital, Sun Yat-sen University, Guangzhou, China; ^2^ The Third Affiliated Hospital, Sun Yat-sen University, Guangzhou, China; ^3^ GE Healthcare Pharmaceutical Diagnostics, Guangzhou, China

**Keywords:** radiomics, computed tomography, progression-free survival, colorectal liver metastases, chemotherapy

Text Correction

In the published article, there was an error. A correction was made to **Author contributions**. In the published original article this section previously stated:

“SY: Formal analysis, Investigation, Writing - original draft. YH: Investigation KXN: Investigation YTL: Methodology, Software. XCT: Conceptualization, Supervision, writing – review and editing. XMP participated in data collection, data analyses, software, and interpretation. All authors contributed to the article and approved the submitted version”.

The corrected version appears below:

“SY, YH and XP are co-authors. SY: Formal analysis, Investigation, Writing - original draft. YH: Investigation KN: Investigation YL: Methodology, Software. XCM: Conceptualization, Supervision, writing – review and editing. XP participated in data collection, data analyses, software, and interpretation. All authors contributed to the article and approved the submitted version”.

The authors apologize for this error and state that this does not change the scientific conclusions of the article in any way. The original article has been updated.

