# Supplementary material for: Association of CT-Based Delta Radiomics Biomarker With Progression-Free Survival in Patients With Colorectal Liver Metastases Undergo Chemotherapy
Source: Front Oncol. 2022 May 27;12:843991. doi: 10.3389/fonc.2022.843991 (PMC9184515; doi:10.3389/fonc.2022.843991)
Supplement: Supplementary file 1 [file DataSheet_1.docx]

**Supplementary material**


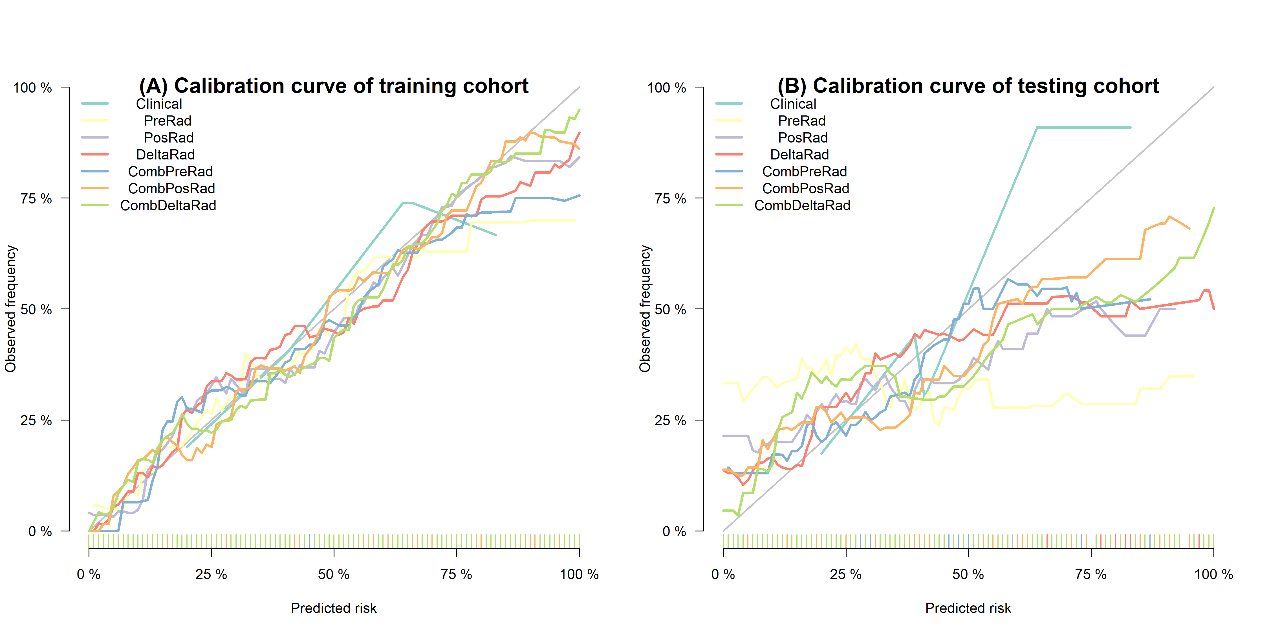


**SuppFigure 1. Calibration curve of training and testing cohort**

**SuppTable 1. P value of Hosmer-Lemeshow test for different models**

| Models | training cohort | testing cohort |
| --- | --- | --- |
| Clinical | <0.001 | 0.206 |
| PreRad | 0.453 | 0.001 |
| PostRad | 0.083 | 0.023 |
| DeltaRad | 0.011 | 0.206 |
| CombPreRad | <0.001 | <0.001 |
| CombPostRad | 0.907 | <0.001 |
| CombDeltaRad | 1.000 | 1.000 |
| Red font indicates p < 0.05 | | |

**SuppTable 2. NRI between different models and clinical model**

| Models | training cohort  NRI [95%CI] | testing cohort  NRI [95%CI] |
| --- | --- | --- |
| PreRad vs clinical | 0.024 [ -0.131 - 0.179 ] | -0.503 [ -0.761 - -0.244 ] |
| PostRad vs clinical | 0.125 [ -0.022 - 0.273 ] | -0.129 [ -0.352 - 0.094 ] |
| DeltaRad vs clinical | 0.253 [ 0.113 - 0.393 ] | 0.042 [ -0.209 - 0.289 ] |
| CombPreRad vs clinical | 0.091 [ -0.024 - 0.206 ] | 0.008 [ -0.180 - 0.195 ] |
| CombPostRad vs clinical | 0.228 [ 0.115 - 0.342 ] | 0.049 [ -0.135 - 0.232 ] |
| CombDeltaRad vs clinical | 0.283 [ 0.171 - 0.395 ] | 0.033 [ -0.188 - 0.254 ] |

**SuppTable 3. IDI between different models and clinical model**

| Models | training cohort  IDI [95% CI] | testing cohort  IDI [95% CI] |
| --- | --- | --- |
| PreRad vs clinical | 0.019 [ -0.105 - 0.143 ] | -0.402 [ -0.611 - -0.193 ] |
| PostRad vs clinical | 0.100 [ -0.018 - 0.219 ] | -0.103 [ -0.283 - 0.077 ] |
| DeltaRad vs clinical | 0.203 [ 0.090 - 0.315 ] | 0.034 [ -0.166 - 0.233 ] |
| CombPreRad vs clinical | 0.073 [ -0.019 - 0.165 ] | 0.006 [ -0.146 - 0.158 ] |
| CombPostRad vs clinical | 0.183 [ 0.091 - 0.274 ] | 0.039[ -0.109 - 0.187 ] |
| CombDeltaRad vs clinical | 0.226 [ 0.136 - 0.316 ] | 0.026 [ -0.152 - 0.205 ] |
